# Beneficial effects of word final stress in segmenting a new language: evidence from ERPs

**DOI:** 10.1186/1471-2202-9-23

**Published:** 2008-02-18

**Authors:** Toni Cunillera, Antoni Gomila, Antoni Rodríguez-Fornells

**Affiliations:** 1 Institució Catalana de Recerca i Estudis Avançats (ICREA), Barcelona, Spain; 2 Dep. Psicologia Bàsica, Facultat de Psicologia, Universitat de Barcelona, Spain; 3 Dep. Psicologia. Universitat Illes Balears, Spain

## Abstract

**Background:**

How do listeners manage to recognize words in an unfamiliar language? The physical continuity of the signal, in which real silent pauses between words are lacking, makes it a difficult task. However, there are multiple cues that can be exploited to localize word boundaries and to segment the acoustic signal. In the present study, word-stress was manipulated with statistical information and placed in different syllables within trisyllabic nonsense words to explore the result of the combination of the cues in an online word segmentation task.

**Results:**

The behavioral results showed that words were segmented better when stress was placed on the final syllables than when it was placed on the middle or first syllable. The electrophysiological results showed an increase in the amplitude of the P2 component, which seemed to be sensitive to word-stress and its location within words.

**Conclusion:**

The results demonstrated that listeners can integrate specific prosodic and distributional cues when segmenting speech. An ERP component related to word-stress cues was identified: stressed syllables elicited larger amplitudes in the P2 component than unstressed ones.

## Background

Segmenting words from fluent speech is a mandatory first step when learning a new language. The difficulty of the task lies in the lack of clear information indicating where a word begins and ends. Following the paradigm introduced by Saffran et al. [1], we exposed adult volunteers to an artificial language while recording event-related brain potentials. After this learning phase, participants were asked to recognize the nonsense words of this artificial language. A specific feature of this language was that no pauses or other potential cues signaling word boundaries were provided. Indeed, the only way to identify the embedded words from the continuous speech stream was by tracking the regular positions of each syllable along the sequence, a computational process (statistical learning) which is operative at the early age of 8 months [1].

Statistical learning is a domain-general mechanism that is involved in a diverse set of sequential situations, such as learning a small artificial grammar [2], sequences of tones [3], and nonsense words from continuous speech [1,4]. Moreover, this learning mechanism appears to be functional not only in audition but also in other sensory modalities such as vision [5] and touch [6]. All in all, the computation of distributional regularities seems to be important for encoding the temporal order and learning the relationships of elements within sequential input.

Word-stress represents another useful cue when learning a language. It can be defined as an abstract phonological property of a word that is more salient or prominent than the other syllables and is established via several different prosodic features, such as pitch, syllable duration and syllable amplitude. In language with a fixed stress pattern, such as Finnish, where stress always falls in the word onset position, stress becomes a reliable segmentation cue [7]. In English, the preponderant word-stress pattern involves stress in the initial syllable of words used in conversational speech; listeners seem to take advantage of this property and apply a segmentation strategy (Metrical Segmentation Strategy) that assumes that every strong syllable in the signal corresponds to the onset of a word [8-11]. When we address this issue in other languages like Spanish, in which stress takes a wider range of positions within words, it is less clear whether or not listeners would benefit from the native language word-stress pattern to segment novel words from a fluent unknown language. In Spanish lexical stress is unpredictable [12]: though penultimate stress is predominant, about one quarter of polysyllabic words have final or antepenultimate stress [13]. As in other languages, lexical stress is signalled by three simultaneous acoustic cues: fundamental frequency (pitch), duration and intensity (see [14,15]) but no vowel reduction is required [16]. Several authors have provided evidences for pitch being the best cue for lexical stress with duration following and, in last place, intensity [13,15-19].

Recently, ERPs have been used to investigate language segmentation [20-23]. Specific ERP components, such as the N1 or the N400, have been proposed as speech segmentation indexes [20]. The N1 seems to be sensitive to word onset perception, whereas the N400 has been considered to indicate the identification of recently segmented words and consequently, it could be understood as the first prelexical brain signature detectable after the speech segmentation task is accomplished. In a previous study we corroborated the finding that the N400 is involved in speech segmentation [24], and in addition, we found that the amplitude of the P2 component was enhanced when a prosodic cue was added on the first syllable of each nonsense word in the stream. Importantly, this increased P2 appeared only for potentially segmented units (trisyllabic nonsense words) and not for syllables presented in random order, even though stress was analogously placed on every third syllable. This distinction clarified the nature of the P2 component in this learning segmentation task reflecting that it was sensitive to an acoustic property (pitch increase) of a unitary group of syllables. To prove the validity of this component in relation to word-stress, further research needs to be conducted in which the placement of a prosodic cue varies within the words.

The main purpose of the present study was to explore the influence of stress as a segmentation cue, its interaction with transitional probabilities, and crucially, the influence of stress on the P2 component when stress is placed on different syllables within a word. If the P2 component is sensitive to the properties of stressed syllables, we should be able to identify the component regardless of the position of the stressed syllable within words.

## Results

### Behavioral Performance

**Figure 1:**
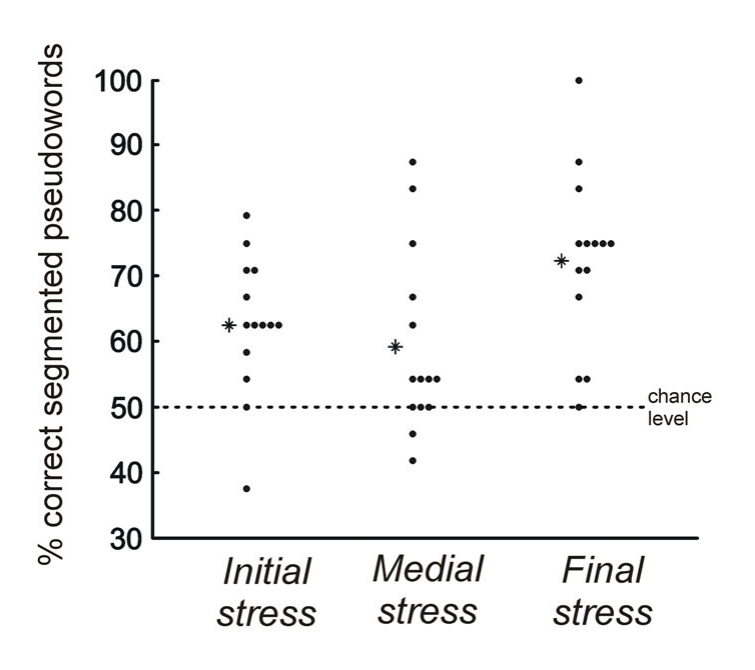
**Percentage of speech segmentation**. Participants' performances on the 2AFC test in each of the three conditions. Black circles represent the score for each subject and asterisks show the mean value for each condition.

For the three different conditions, the percentage of correctly segmented pseudo-words was: Initial stress: 62.5 ± 10.6%; Medial stress: 59.2 ± 13.9%; and Final stress: 72.3 ± 13.4% (Figure 1). All mean percentages were different from chance (50%) (initial stress: *t*(13) = 4.42, P < 0.01; medial stress: *t*(13) = 2.47, P < 0.05; and final stress: *t*(13) = 6.22, P < 0.001). The ANOVA revealed that performance in the three conditions differed from each other (F(2,26) = 4.44, P < 0.05; η_*p*_^2 ^= 0.26). Pairwise comparisons showed that the only significant difference was found between the final and medial stress conditions (*t*(13) = -3.13, P < 0.01, effect size: *d *= 0.96; initial vs. medial, t(13) < 1, *d *= 0.26; final vs. initial, t(13) = -1.96, P = 0.07, *d *= 0.81).

We further conducted a chi-square test in order to ensure that the observed differences among conditions were reliable^1^. We proceeded by first identifying how many participants in each condition performed better than expected by chance [7]. Chance level was determined using a binomial test (with P < 0.05, in a 24-item test). Accordingly, the performance at or above 66.67% is significantly better than chance. In the initial stress condition, 5 out of 14 (35.7%) participants performed above chance; in the medial stress condition, there were 4 out 14 participants (28.6%) that performed above chance; and finally, in the final stress condition, 11 out 14 participants (78.6%) performed above the chance level. A chi-square test was then calculated to compare between conditions. The results of the chi-square test revealed that the difference between the final and the medial stress conditions was significant (χ^2^(1) = 7.04, P < 0.01), as well as the difference between the initial and the final stress condition (χ^2^(1) = 5.25, P < 0.05). Finally, the chi-square revealed that the difference between the initial and medial stress conditions did not reach statistical significance (χ^2^(1) = 0.16, P > 0.05). In sum, the behavioral results showed that participants' performances were better when stress fell on the word final syllable.

### ERPs

**Figure 2:**
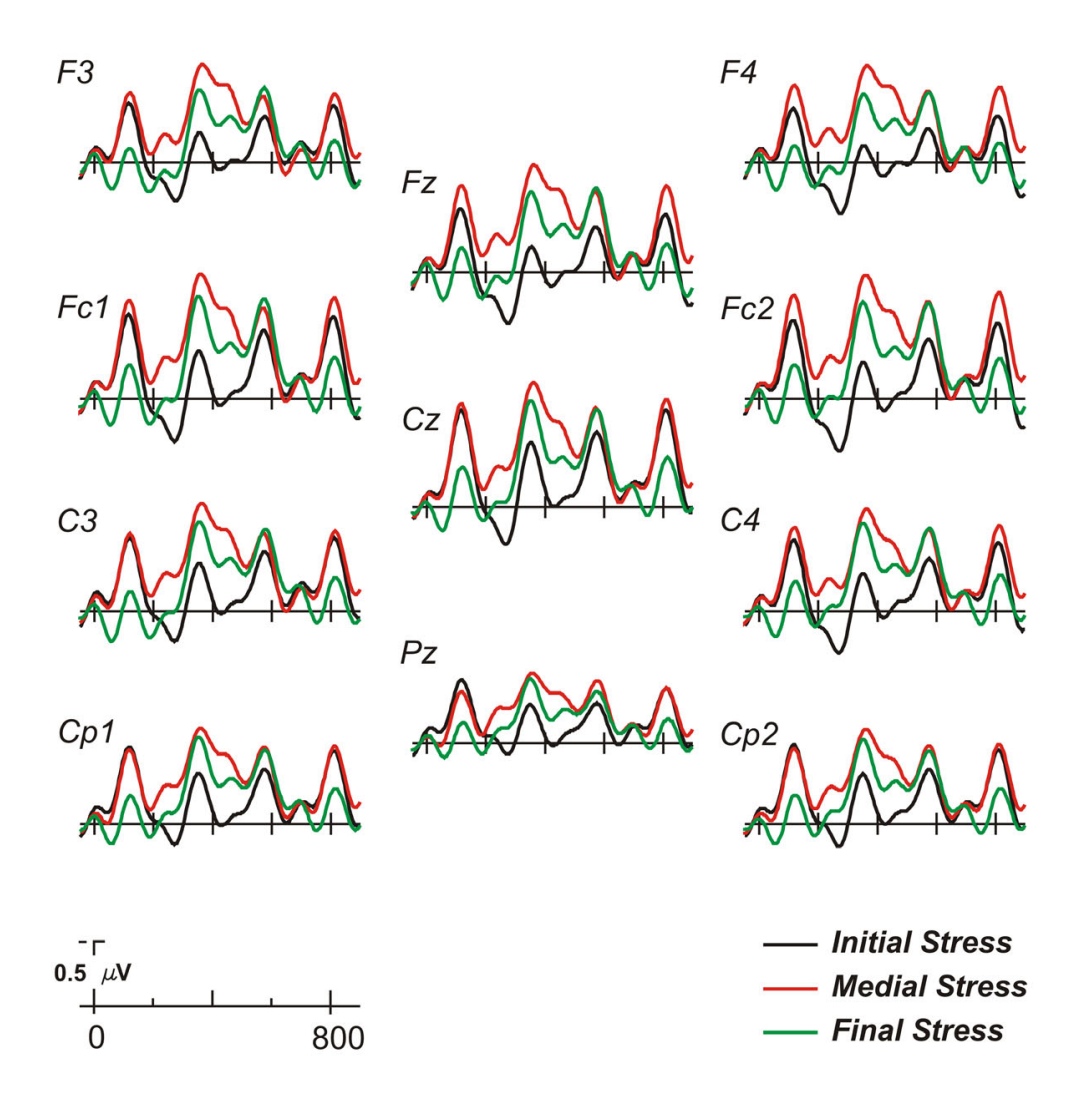
**General ERP effects**. Grand average ERPs (n = 14) elicited by words of the three different conditions (initial, medial, and final stress). Different electrode positions at central and parasagittal locations are shown.

**Figure 3:**
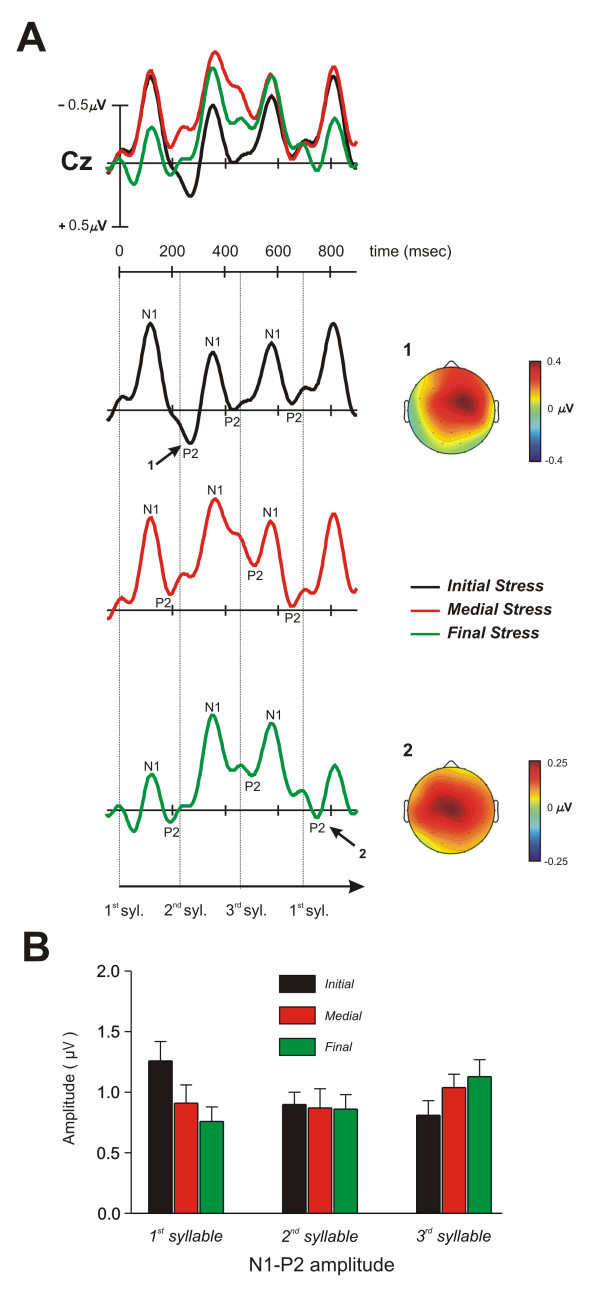
**Modulation of the P2 component in each syllable and condition**. A. Grand average ERPs for words with initial, medial, and final stress-conditions shown separately at a midline (Cz) electrode position (upper left side). A very similar morphology of the P2 component for initial and final stress conditions can be observed in the topographical maps (upper right side). The difference waveform of the mean amplitude at the corresponding P2 time-window is shown for the first and third syllable for the initial and final stress condition, respectively: 1. initial stress (initial stress minus final stress conditions); 2. final stress (final stress minus initial stress conditions). Isovoltage mapping with spherical spline interpolation was used to depict the scalp distribution of the difference waveforms. No differences were found in the scalp distribution of both P2 components [Condition (first syllable, initial stress; last syllable, final stress) x electrode locations (29 electrodes): F(28, 364) = 1.01; P > 0.41]. B. The mean amplitude (standard error of the mean) for the peak-to-peak N1-P2 component at a midline (Cz) location for each syllable and condition.

Grand average ERPs for the initial, medial, and final stress conditions are depicted in Figures 2 and 3. The N1-P2 complex of the auditory evoked components was clearly identified at central electrodes for each condition (see Figure 2 and 3). These components were followed by a broadly distributed negativity (range 300–500 msec) particularly for the medial stress condition. Moreover, the N1-P2 complex was repeatedly observed throughout the epoch starting approximately every 232 msec, which corresponded to the syllable duration. The ERP waveforms for each condition were characterized by an enhanced P2 component elicited by the stressed syllable. The clearest effect of stress on this component was observed mainly for the first and third syllables of the initial and final stress condition, respectively (see Figure 3). This positivity was also observed in the medial stress condition for the second syllable, although the effect was less marked because it overlapped with a negative deflection.

The repeated measures ANOVA for the N1 peak amplitude including stress-condition (initial, medial, and final) and syllable-position (first, second, and third) found no statistically significant differences. However, a significant trend was found for the interaction between the two factors (F(4,52) = 2.29, ε = 0.6, P = 0.072, η_*p*_^2 ^= 0.15) probably due to the reduced amplitude of the N1 in the first-syllable of the final stress condition. Further pairwise comparisons applied only to the initial syllable confirmed that the amplitude of the N1 was significantly reduced only when the final stress condition was compared to the medial stress condition (*t*(13) = -2.48, P < 0.03, *d *= 0.76; initial stress vs. final stress, *t*(13) = 1.67, P > 0.1, *d *= 0.64; initial stress vs. medial stress, *t*(13) = 0.1, P > 0.9 *d *= 0.02). For the P2 peak amplitude, an effect of syllable-position was found (F(2,26) = 6.03, ε = 0.77, P < 0.01, η_*p*_^2 ^= 0.32), indicating that the P2 amplitude was larger in the first syllable in spite of the stress condition (see Table 1).

**Table 1:** Amplitude (in μV ± s.e.m.) of the N1, P2 and N1-P2 components for each syllable and condition (Cz location). Bold values correspond to the crucial syllable-position for the initial, medial, and final Stress-conditions.

	*Syllable-position*		
*Stress condition*	1^st ^syllable	2^nd ^syllable	3^rd ^syllable
	μV ± *s.e.m*.	μV ± *s.e.m*.	μV ± *s.e.m*.
**N1**			
initial	-0.86 ± *0.17*	-0.68 ± *0.15*	-0.67 ± *0.15*
medial	-0.87 ± *0.13*	-1.15 ± *0.20*	-0.87 ± *0.14*
final	-0.47 ± *0.14*	-0.89 ± *0.14*	-0.84 ± *0.13*
**P2**			
initial	0.40 ± *0.16*	0.22 ± *0.15*	0.14 ± *0.09*
medial	0.04 ± *0.13*	-0.29 ± *0.18*	0.17 ± *0.10*
final	0.28 ± *0.18*	-0.04 ± *0.12*	0.29 ± *0.09*
**N1-P2**			
initial	**1.26 **± *0.16*	0.90 ± *0.10*	0.81 ± *0.12*
medial	0.91 ± *0.15*	**0.87 **± *0.16*	1.04 ± *0.11*
final	0.76 ± *0.12*	0.86 ± *0.12*	**1.13 **± *0.14*

Most probably, the reduction in amplitude for the N1 component observed in the first syllable (final stress) was due to the overlap of the positivity developed in the last syllable where the stress is located. Notice that the duration of each word is 696 msec, and therefore the pre-stimulus baseline was influenced by the components elicited in the last syllable. In order to avoid the influence of this pre-stimulus baseline in our statistical analysis, we opted for a peak-to-peak analysis (see below), which is independent of the baseline chosen.

When the ANOVA was conducted on the N1-P2 peak-to-peak amplitude measure, the statistical results did not reveal a main effect of either the stress-condition (F(2,26) < 1, η_*p*_^2 ^= 0.03) or the syllable-position (F(2,26) = 1.23, ε = 0.8, P > 0.3, η_*p*_^2 ^= 0.09), although a significant interaction was found (F(4,52) = 2.96, ε = 0.78, P < 0.05, η_*p*_^2 ^= 0.19). This effect reflects a selective increase in the amplitude for the stressed syllable in the initial stress condition (first-syllable vs. third-syllable: *t*(13) = 3.68, P < 0.01, *d *= 0.86; see also Table 1), as well as in the third stressed syllable in the final stress condition (third-syllable vs. initial-syllable: *t*(13) = -2.25, P < 0.05, *d *= 0.78; third-syllable vs. second-syllable: *t*(13) = -2.01, P < 0.06, *d *= 0.56). For the medial stress condition, however, the largest peak-to-peak amplitude was found on the third syllable, but no statistical differences were observed between the first, second and third syllable (see Figure 3b). This P2 component was characterized by a mid-central scalp distribution, which was similar in both initial and final stress conditions (see topographical maps of the difference waveforms in Figure 3a).

## Discussion

In the current study we evaluated the effect of a prosodic cue on speech segmentation using on-line ERP measures. We recorded ERPs to artificial language (nonsense words) with the presence of a stress cue in different syllable positions. In addition, a statistical cue was always present, which corresponded to the transitional probability signaling the three syllable unit (a nonsense word).

Although a reduction in the N1 peak amplitude component was found for word onset in the final stress-condition, this effect should be interpreted only as the result of the overlap of the stress-P2 component elicited by the last syllable. Due to the specific structure of these continuous sound streams, the pre-stimulus baseline condition is always affected by the components elicited in the last syllable. For example, the same overlap occurs for the N1 component elicited in the second syllable in the initial stress-condition in which the amplitude is reduced due to the larger amplitude of the previous stress-related P2 component. In this regard, the peak-to-peak measures proved to be a more reliable method for analyzing the current ERP data [25].

Increased amplitude in the P2 component was found for stress syllables in all three conditions, although it was more discernible in the first and third stressed syllable of segmented words. The behavioral results clearly showed that participants segmented the stream better when stress fell on the last syllable than when it fell either on the first or in the middle syllable of the nonsense words. This pattern of behavioral results, however, might have been influenced by the difficulty of the test, as part-word foils were used instead of non-words. Besides, in order to match test items, word-stress was removed from all test items^3^. Overall, the statistical power might have been reduced in the test-phase of the present design due to this manipulation. Further studies might need to address if the initial stress condition would have yielded a better segmentation rate when using a different type of design in the phase test.

In real language acquisition, segmentation cues are never applied alone. Rather, it seems plausible that the combination of several different cues would yield a significant improvement in solving the task of identifying embedded units. Few studies have directly addressed the issue of how the combination of different types of cues facilitates speech segmentation and most of those performed have tested different cues during competition between them  [26-30]. In a similar experiment to the one presented here, Saffran et al. [4] introduced a stress cue (syllable lengthening) on either the first or the last syllable in two different conditions. A facilitatory effect of stress was observed only when it was placed at the end of the word, suggesting that participants used their tacit knowledge of word-final lengthening to segment the speech stream. In our study, we observed a reduction in the percentage of segmented words when stress was introduced in the penultimate syllable. This result suggests that the non-coincidence of the statistical and prosodic cues might elicit conflict in this condition. In a similar vein, Thiessen and Saffran [30] found that 9-month-old infants exposed to conflicting statistical and stress information in an artificial language stream were more reliant on stress than on statistical cues in order to segment the words. The infants seemed to treat stressed syllables as word onset even in the case where it led them to mis-segment the words from the stream. Importantly, the reverse result was obtained with 7-month-olds, who relied more on the statistical information than on the rhythmic pattern of the language. In a recent study with adults using a crossmodal-priming paradigm, Mattys et al. [29] proposed the existence of weights for the different speech segmentation cues and situated stress in the last tier of this hierarchy. As stated in the introduction, Spanish does not have a fixed stress pattern, though penultimate syllable stress predominates^2^. It is therefore possible that listeners in Spanish do not take advantage of this cue when segmenting words from an unknown language. If listeners had been able to exploit this medial stress pattern, a larger segmentation rate would have been expected as they might have taken advantage of this prosodic pattern and the statistical information contained in the boundaries. The pattern observed in the present study clearly indicates that they did not exploit this medial stress pattern. Hence, it is likely that listeners do not rigidly use the tacit knowledge of their native language about the word-stress pattern to learn words from a new language; rather, they might use word-stress as a cue but in a more flexible fashion (in regard to its position in a word). Thus, this result might reflect that the learning system is flexible enough to bypass the default stress language pattern and to focus on the convergence of the segmentation cues (pitch and transitional probabilities). In this regard, this result does not agree with a hierarchical system, whereby reliance on some cues is greater than reliance on others. Instead, a more flexible and dynamic system might be able to adjust to the most advantageous learning pattern.

The results obtained in the two conditions where word-stress indicates word offset/onset (e.g., word-stress placed at the final syllable) suggest that the combination of stress and other distributional cues, like transitional probabilities, seems to slightly facilitate the localization of the embedded words in the streams. Therefore, the present results are suitable with two possible explanations: either (i) the listeners are not able to track the different segmentation cues at the same time if those cues are not located in the same syllable boundary (word-stress middle syllable; transitional probability at the end/onset of the word) or (ii) there is a strong facilitation when two types of segmentation cues coincide. Both interpretations could be unified if we consider that when two segmentation cues coincide, the attentional focus coincides as well (being localized in the same syllable boundary). On the other hand, when two cues do not coincide, the attentional focus might be more disperse and consequently the listener's performance might decrease.

Additionally, our results corroborate the notion that learners pay more attention to the ends of the words [31,32]. In a recent study with adults engaged in a repetition detection task, Endress et al. [33] found that participants only succeeded in generalizing repetition-based structures when the repetitions were located at the final edges of sequences and were unable to do so when repetition was sequence-internal. For the authors, their results reflected a benefit of processing 'perceptual salience' of certain kinds of structures.

A possible explanation for our pattern of results is that the P2 component may be mostly related with the distributional properties of stressed syllables. Although the placement of the stressed syllable varied in the three conditions of the experiment, all the language streams resembled each other, being made up of concatenating trisyllabic words with the same stress pattern. This produced the critical characteristic that a stressed syllable was heard in every three syllables, regardless of the condition, and therefore, the stress pattern introduced in the stimulation could act as another distributional cue useful for indicating word offset-onset.

Considering the previous interpretations, the P2 component elicited by stressed syllables could be interpreted as an attentional cue related to the perceptual learning process itself. For instance, the involvement of attention is important in experiments that reported increased P2 sensitivity to learning [23,34-36]. In regard with language learning, De Diego et al. [23] observed a increased P2 component that positively correlated with listener's perception of initial-final syllable grouping (i.e., with abstract rule-learning). Interestingly, the authors hypothesized that the P2 reflected the reallocation of attention, which was postulated as necessary for learning non-adjacent dependencies between syllables.

Data obtained from somatosensory and visual modalities in various perceptual discrimination tasks support the view that attention is involved in the neurophysiologic changes observed during learning [37-42]. Thus, word-stress seems to serve as a reliable cue in attentional capture for facilitation of speech analysis [43]. In behavioral studies, in which a phoneme monitoring task was used, it has been observed that word-stress serves as an attentional factor which facilitates the target (phoneme) detection [44-46], which supports the present observations on the P2 component and the exploitation of speech segmentation cues. But recently, in a study of auditory scene analysis where attention was manipulated in two different conditions, the authors found that in spite of attention, the amplitude of the P2 component correlated with behavioral judgments of streaming, which measures the capacity of listeners to perceive two different pure-tone streams [47]. The main finding of this study, however, was the fact that the P2 component appeared to be sensitive to auditory perceptual discrimination. Other studies have found that pitch modulated the P2 component [48], suggesting that pitch, as a stress mark, is used effectively in spoken word recognition [49].

## Conclusion

The present study shows that adults are able to integrate specific prosodic and statistical cues when segmenting speech. The electrophysiological results showed that stressed syllables elicited a larger amplitude on the P2 component than non-stressed syllables. Finally, the behavioral results showed that segmentation was improved when word-stress was found on the last syllable of a word. We propose that this P2 modulation is associated with the underlying learning mechanism in charge of processing the distributional properties of stressed syllables in a continuous speech stream.

## Methods

### Participants

Fourteen volunteers (4 males) who were undergraduate Psychology students at the University of Barcelona received extra course credits for their participation. All participants [mean age 21 ± 3.8 (SD)] were right-handed Spanish-Catalan speakers and reported no hearing deficits or knowledge of neurological impairments. Three participants' data were omitted from the analysis due to excessive eye movement and finally the data from 14 participants was analyzed. In accordance with the procedures of the institutional local ethics committee (University of Barcelona), written consent was obtained from all participants.

### Stimuli and procedure

Nine language streams were created following the structure described by Saffran et al. [1]. A pool of 59 different consonant-vowel syllables was combined in a way that twelve different syllables were used each time to create a language stream. This process was performed twice, and finally nine streams were generated.

Each stream was made by concatenating 4 trisyllabic nonsense words that were repeated 192 times, each in a pseudorandom order with no acoustic pauses between them and with the stipulation that the same word never occurred twice in a row. The duration for each syllable was set at 232 msec, so each word lasted 696 msec, yielding a language stream of 8 minutes and 54 seconds. For all the streams the transitional probability within a trisyllabic grouping was always 1.0, whereas for syllables spanning unit boundaries it was 0.33. An orthographic example of the speech stream is the string "*pirutabagolitokudabagoligukibo*...", in which "piruta", "bagoli", "tokuda", and "gukibo" are the possible words to be segmented.

After the creation of the basic language streams, a pitch increase of 20 Hz (hereafter referred to as "stress") was added to one syllable in a fixed position of each trisyllabic word in the stream. Consequently, of every three syllables one was stressed during the stream (e.g., "*PIrutaBAgoliTOkudaBAgoliGUkibo*..."). This provided an inventory of 27 language streams, which were divided into three conditions depending on whether the stress was positioned on the initial, medial or final syllable within the words. The speech synthesizer MBROLA [50] (with a Spanish male diphone database at 16 kHz.) was used to synthesize all the auditory stimulation and afterwards the Cooledit software was used to equate the length of the different streams into a msec precision.

Note that in the initial stress as well as in the final stress conditions the streams contained an acoustic indication of word boundaries but not in the medial stress condition. The acoustic feature provided by increasing the pitch of a selective syllable within a word allowed us to have a direct comparison between conditions because the length of the syllables was equal in all conditions.

Finally, test items were created in order to test participants' segmentation performance (percentage of segmented words). For all conditions, items consisted of the four words composing each stream and part-words made by concatenating the last two syllables of a word and the first one of another, or the last syllable of a word and the first two syllables of another. Importantly, stress was removed from the test items to match words and part-words.

Participants took part in a single two-hour ERP recording session, in which 3 initial, 3 medial, and 3 final stressed language streams (the 9 basic streams) were presented in a pseudo-random order and where no more than two successive streams of the same condition occurred. They were required to listen carefully to a "Martian" language and asked to identify the words that formed it. They were also instructed to refrain from making eye movements and to reduce blinking while hearing the streams. The stress position for each stream was balanced across participants.

At the end of each stream a two alternative forced choice (2AFC) test was administered to the participants to determine whether they were able to identify the words heard just before in the stream. The test comprised 8 pairs of test items (words – part-words) presented in random order, in which each of the 4 words forming the stream was presented twice. The overall score for each condition was computed over a maximum of 24 possible correct responses. After hearing each test item pair, the participants were asked to decide, by pressing one button corresponding to the first or second item of the pair, which item of the stream was heard as a word. The presentation of the items of a pair was separated by 500 msec of silence.

After each stream and its subsequent test participants were allowed to rest. After a brief pause, the next stream was presented.

### Electrophysiological Recording

The ERPs were recorded from the scalp using tin electrodes mounted in an electrocap (ECI) and located at 29 standard positions. The data was referenced on-line to the outher canthus of the right eye and off-line to the average of the mastoid recordings. Vertical eye movements were monitored with an electrode at the infraorbital ridge of the right eye. Electrode impedances were kept below 3 KOhm. The electrophysiological signals were filtered on-line with a bandpass of 0.01–50 Hz (half-amplitude cutoffs) and digitized at a rate of 250 Hz. Trials with a base-to-peak electro-oculogram (EOG) amplitude of more than 50 μV, amplifier saturation, or a baseline shift exceeding 200 μV/s were automatically rejected off-line.

### Data analyses

Stimulus-locked ERPs were averaged for epochs of 1024 msec starting 100 msec prior to the stimulus. After averaging, epochs were filtered with a bandpass of 1–6 Hz, because this filtering procedure facilitates the peak-to-peak measurement of the P2 components along the epoch for all experimental conditions. Separate ERPs from individual volunteers were computed for each condition (initial, medial, and final stress position streams) at the Cz electrode location. N1 and P2 peak-amplitude values elicited for each syllable and conditions (as well as the peak-to-peak amplitude difference between the P2-N1 components) were measured. These values were submitted separately to a repeated measures analysis of variance (ANOVA) including two within-subject factors: stress-condition (initial, medial, and final) and syllable-position (first, second, and third). The peak latencies of the N1 component were located in the grand average at 118, 362, and 576 msec after word onset for the initial, medial, and final stress syllable, respectively. For all participants and conditions the N1 was sought at 0–268, 212–512, and 426–726 msec time windows. For the P2 component the peak was found at 270, 510, and 748 msec after word onset, and the peak was sought at 120–420, 360–660, and 598–898 msec time windows, respectively.

Additionally two-tailed t-tests were used for pairwise comparisons. For all statistical effects involving two or more degrees of freedom in the numerator, the Greenhouse-Geisser epsilon was applied when necessary to correct for violations of the sphericity assumption [51]. The exact *p*-value after the correction is reported.

Peak-to-peak analysis was selected because (i) it provided an independent measure which was not affected by previous positive or negative deflections [25], and (ii) the N1-P2 peak-to-peak difference provided a reliable measure of the P2 component when modulated by stress.

## Authors' contributions

TC carried out the ERPs recording and analysis, performed the statistical analysis and finally drafted the manuscript. AG participated in the design of the study and helped to draft the manuscript. ARF participated in the design and its coordination, supervised the statistical analysis and the drafting of the manuscript. All authors read and approved the final manuscript.

## Footnotes

1. We opted for reanalyzing the data using a chi-square test because this non-parametric test is less sensitive to biases introduced in the data when some participants perform extremely well or poorly. These extreme values notably affect the mean score and the standard deviation and, consequently, the t-test comparisons, masking potential differences between groups (an increase of the probability of the Type II errors) [52].

2. In the case of trisyllabic words with the same syllabic pattern as the ones used in the experiment (CVCVCV; C: consonant; V: vowel), the stress is found 12.85% in the first syllable, 76.16% in the second syllable and 10.99% in the last syllable (percentage calculated from LEXESP database which contains 3,222 trisyllabic CVCVCV words, which represents a 0.064% of the total amount of tokens in the database). Similar percentages are found when taking into account trisyllabic words of all kind of syllabic patterns (first syllable: 5.47%; second syllable: 75.69%; third syllable: 18.84%; trisyllabic words are the 0.76% of tokens in the database, which contains 5,020,930 word items).

3. Word-stress was removed from the test foils in order the equate words with part-words, otherwise participants could have responded based only on the stress pattern (which would be fixed in words and variable in part-words). In a recent study on speech segmentation with a similar paradigm than the one used in the present study, the authors used in the test phase either (i) auditory presented items in one experiment (in which word-stress was also removed from the items) or (ii) visually presented items in another experiment [53]. The rationale of the authors was that using visual items allowed participants, when reading the test items, to generate the auditory stored representations of words segmented in the learning phase. Interestingly, when the authors compared the results of the two experiments no effect of modality (auditory vs. visually) was encountered, indicating that participants recognized word-like units in the test phase in spite of the absence of word-stress.

## References

[B1] Saffran JR, Aslin RN, Newport EL (1996). Statistical learning by 8-month-old infants. Science.

[B2] Gomez RL, Gerken L (1999). Artificial grammar learning by 1-year-olds leads to specific and abstract knowledge. Cognition.

[B3] Saffran JR, Johnson EK, Aslin RN, Newport EL (1999). Statistical learning of tone sequences by human infants and adults. Cognition.

[B4] Saffran JR, Newport EL, Aslin RN (1996). Word segmentation: The role of distributional cues. J Mem Lang.

[B5] Fiser J, Aslin RN (2002). Statistical learning of higher-order temporal structure from visual shape sequences. Journal of Experimental Psychology-Learning Memory and Cognition.

[B6] Conway CM, Christiansen MH (2005). Modality-constrained statistical learning of tactile, visual, and auditory sequences. Journal of Experimental Psychology-Learning Memory and Cognition.

[B7] Vroomen J, Tuomainen J, de Gelder B (1998). The roles of word stress and vowel harmony in speech segmentation. J Mem Lang.

[B8] Cutler A, Carter D (1987). Metrical structure of initial syllables in English. Journal of the Acoustical Society of America.

[B9] Cutler A, T.M.Altmann G (1990). Exploiting prosodic probabilities in speech segmentation. Psycholinguistic and Computational Perspectives.

[B10] Cutler A, Butterfield S (1992). Rhythmic cues to speech segmentation - Evidence from juncture misperception. J Mem Lang.

[B11] Cutler A (1994). Segmentation problems, rhythmic solutions. Lingua.

[B12] Navarro Tomás T, científicas CSI (1965). Manual de pronunciación española.

[B13] Quilis A, Ariel  (1984). Métrica española.

[B14] Enriquez E, Casado C, Santos A (1989). La percepción del acento en español. Lingüística Española Actual.

[B15] Llisterri J, Machuca MJ, de la Mota C, Riera M, Ríos A, Solé MJ, Recasens D and Romero J (2003). The perception of lexical stress in Spanish.

[B16] Ortega-Llobaria M, Díaz-Campos M (2006). Phonetic cues to stress and accent in Spanish.

[B17] Gili-Gaya S, Gredos  (1981). Elementos de fonética general.

[B18] Quilis A (1971). Caracterización fonética del acento en español. Travaux de Linguistique et de Litterature.

[B19] Quilis A, Gredos  (1981). Fonética acústica de la lengua española.

[B20] Sanders LD, Newport EL, Neville HJ (2002). Segmenting nonsense: an event-related potential index of perceived onsets in continuous speech. Nat Neurosci.

[B21] Sanders LD, Neville HJ (2003). An ERP study of continuous speech processing I. Segmentation, semantics, and syntax in native speakers. Cogn Brain Res.

[B22] Sanders LD, Neville HJ (2003). An ERP study of continuous speech processing II. Segmentation, semantics, and syntax in non-native speakers. Cogn Brain Res.

[B23] De Diego-Balaguer R, Toro JM, Rodriguez-Fornells A, Bachoud-Levi AC (2007). Different neurophysiological mechanisms underlying word and rule extraction from speech. PLoS ONE.

[B24] Cunillera T, Toro JM, Sebastian-Galles N, Rodriguez-Fornells A (2006). The effects of stress and statistical cues on continuous speech segmentation: An event-related brain potential study. Brain Research.

[B25] Picton TW, Bentin S, Berg P, Donchin E, Hillyard SA, Johnson R, Miller GA, Ritter W, Ruchkin DS, Rugg MD, Taylor MJ (2000). Guidelines for using human event-related potentials to study cognition: Recording standards and publication criteria. Psychophysiology.

[B26] Johnson EK, Jusczyk PW (2001). Word segmentation by 8-month-olds: When speech cues count more than statistics. J Mem Lang.

[B27] Jusczyk PW, Cutler A, Redanz NJ (1993). Infants preference for the predominant stress patterns of English words. Child Development.

[B28] Mattys SL, Jusczyk PW (2001). Phonotactic cues for segmentation of fluent speech by infants. Cognition.

[B29] Mattys SL, White L, Melhorn JF (2005). Integration of multiple speech segmentation cues: A hierarchical framework. Journal of Experimental Psychology-General.

[B30] Thiessen ED, Saffran JR (2003). When cues collide: Use of stress and statistical cues to word boundaries by 7-to 9-month-old infants. Developmental Psychology.

[B31] Echols CH, Newport EL (1992). The role of stress and position in determining first words. Language Adquisition.

[B32] Slobin DI (2006). Cognitive prerequisites for the development of grammar. Studies of child language development.

[B33] Endress AD, Scholl BJ, Mehler J (2005). The role of salience in the extraction of algebraic rules. Journal of Experimental Psychology-General.

[B34] Atienza M, Cantero JL, Dominguez-Marin E (2002). The time course of neural changes underlying auditory perceptual learning. Learn Mem.

[B35] Tremblay K, Kraus N, Mcgee T, Ponton C, Otis B (2001). Central auditory plasticity: Changes in the N1-P2 complex after speech-sound training. Ear Hear.

[B36] Tremblay KL, Kraus N (2002). Auditory training induces asymmetrical changes in cortical neural activity. J Speech Lang Hear Res.

[B37] Ahissar M, Hochstein S (1993). Attentional control of early perceptual-learning. Proc Natl Acad Sci U S A.

[B38] Ahissar M, Hochstein S (2000). The spread of attention and learning in feature search: effects of target distribution and task difficulty. Vision Res.

[B39] Joseph JS, Chun MM, Nakayama K (1997). Attentional requirements in a 'preattentive' feature search task. Nature.

[B40] Recanzone GH, Merzenich MM, Jenkins WM, Grajski KA, Dinse HR (1992). Topographic reorganization of the hand representation in cortical area 3B of owl monkeys trained in a frequency-discrimination task. J Neurophysiol.

[B41] Recanzone GH, Jenkins WM, Hradek GT, Merzenich MM (1992). Progressive improvement in discriminative abilities in adult owl monkeys performing a tactile frequency discrimination task. J Neurophysiol.

[B42] Recanzone GH, Schreiner CE, Merzenich MM (1993). Plasticity in the frequency representation of primary auditory-cortex following discrimination-training in adult owl monkeys. J Neurosci.

[B43] Wang JT, Friedman D, Ritter W, Bersick M (2005). ERP correlates of involuntary attention capture by prosodic salience in speech. Psychophysiology.

[B44] Pallier C, Sebastian-Galles N, Felguera T, Christophe A, Mehler J (1993). Attentional allocation within the syllabic structure of spoken words. J Mem Lang.

[B45] Pitt MA, Samuel AG (1990). The use of rhythm in attending to speech. Journal of Experimental Psychology-Human Perception and Performance.

[B46] Shields JL, Mchugh A, Martin JG (1974). Reaction-time to phoneme targets as a function of rhythmic cues in continuous speech. Journal of Experimental Psychology.

[B47] Snyder JS, Alain C, Picton TW (2006). Effects of attention on neuroelectric correlates of auditory stream segregation. Journal of Cognitive Neuroscience.

[B48] Friedrich CK, Alter K, Kotz SA (2001). An electrophysiological response to different pitch contours in words. Neuroreport.

[B49] Friedrich CK, Kotz SA, Friederici AD, Alter K (2004). Pitch modulates lexical identification in spoken word recognition: ERP and behavioral evidence. Cogn Brain Res.

[B50] Dutoit T, Pagel N, Pierret F, Bataille O, van der Vreken O (1996). The MBROLA project: Towards a set of high-quality speech synthesizers free of use for non-commercial purposes.

[B51] Jennings JR, Wood CC (1976). Epsilon-adjustment procedure for repeated-measures analyses of variance. Psychophysiology.

[B52] Zimmerman DW (1994). A note on the influence of outliers on parametric and nonparametric tests. The Journal of General Psychology.

[B53] Shukla M, Nespor M, Mehler J (2007). An interaction between prosody and statistics in the segmentation of fluent speech. Cognitive Psychology.

